# Vitamin D deficiency in adolescents in a tier 4 psychiatric unit

**DOI:** 10.1192/pb.bp.115.053298

**Published:** 2017-06

**Authors:** Neil F. Stewart, Simon N. Lewis

**Affiliations:** 1 Whittington Health NHS Trust, London, UK

## Abstract

**Aims and method** To review the current clinical practice and guidelines for testing and treating vitamin D deficiency in adolescents admitted to a tier 4 adolescent psychiatric unit in north London. The blood test results of 56 patients admitted between 2012 and 2014 were examined to determine whether vitamin D levels had been tested. For those individuals who were tested for vitamin D, results were analysed by gender and ethnicity.

**Results** Of 56 patients admitted, 48% were tested for vitamin D deficiency and in 81.5% of cases we uncovered deficiency or severe deficiency; 18.5% had the minimum levels of vitamin D for bone health as per our trust guidelines.

**Clinical implications** Adolescents within tier 4 adolescent mental health services may be at higher risk of vitamin D deficiency and so assessment of vitamin D levels should be considered as part of a standard physical health review for this group of young people.

It is well known that vitamin D plays a role in bone health. In addition, vitamin D receptors have been found in several areas of the brain and are reported to be involved in neuroimmuno-modulation, neuroprotection, neuro-plasticity and brain development.^1^ Given that some of these receptors are found in brain areas implicated in mental illnesses such as depression,^1^ it is plausible that vitamin D and/or vitamin D deficiency have a role in the pathogenesis of mental illness.

Existing guidelines identify risk factors for vitamin D deficiency^2–4^ (details available from the authors on request), although there is little that relates specifically to adolescents or individuals within mental health settings. We hypothesised that patients in a tier 4 adolescent psychiatric unit in north London may be at increased risk of vitamin D deficiency, potentially having had reduced exposure to sunlight as a result of their psychiatric presentations. Linked to this, according to data from the UK National Census in 2011,^5^ there is a higher proportion of individuals with increased skin pigmentation (black and minority ethnic (BME)) compared with the national average within inner London schools, which may reflect general population trends in urban areas.

## Aim

We examined the blood test results of all patients admitted to Simmons House, a tier 4 adolescent psychiatric unit, to review our management of individuals with vitamin D deficiency, make recommendations for future practice and identify areas where further study is needed.

## Method

The blood test results of patients admitted to the unit between 2012 and 2014 were examined after a clinical observation that a number of patients had vitamin D deficiency. Patient notes were checked to determine gender, ethnicity, whether they were tested for vitamin D levels and the result of the test. The results were classified according to local laboratory reference ranges at the Whittington Hospital NHS Trust in London: <25 nmol/L severe deficiency, 25–50 nmol/L deficiency and 50 nmol/L minimal vitamin D levels for bone health. The results were also mapped to other guidelines, such as National Institute for Health and Care Excellence (NICE) guidelines (<25 nmol/L ‘low vitamin D status’) and those outlined by the Endocrine Society and Society for Adolescent Health and Medicine (>75 nmol/L ‘adequate’).^4,7^

## Results

Of 56 patients on the unit between 2012 and 2014, 48% (*n* = 27) were tested for vitamin D deficiency. According to local laboratory reference ranges (Whittington Hospital NHS Trust), 81.5% (*n* = 22) were deficient or severely deficient (40.7% deficient and 40.7% severely deficient) and 18.5% had the minimum levels for bone health. Applying the NICE guidelines, 40.7% of patients had low vitamin D status. No individuals tested had levels of >75 nmol/L (a level recommended in other guidelines).^4,7^ The results are summarised in Table 1.

**Table 1:** Vitamin D levels in the tested patient sample

	Patients *n*	Tested for vitamin D levels (%)	Deficient or severely deficient^a^ (%)	Minimum level of vitamin D for bone health^b^ (%)	Vitamin D at > 75 nmol/L
Male	14	8 (57)	6 (75)	2 (25)	0 (0)

Female	42	19 (45.2)	15 (79)	4 (21)	0 (0)

White	39	18 (46.1)	13 (72.2)	5 (27.8)	0 (0)

BME	17	9 (52.9)	9 (100)	0 (0)	0 (0)

Total	56	27 (48)	22 (81.5)	5 (18.5)	0 (0)

BME, Black and minority ethnic.

a. <50 nmol/L

b. >50 nmol/L

### Results by ethnicity

Nearly half of adolescents who were White were tested (46.1%, 18/39), and 72.2% (*n* = 13) were deficient or severely deficient in vitamin D. In individuals from BME groups, who were potentially at higher risk of vitamin D deficiency due to increased skin pigmentation, 52.9% (9/17) were tested for vitamin D levels and 100% were deficient or severely deficient. There was no significant statistical association between ethnicity and vitamin D deficiency (χ^2^ = 3.07, *P* = 0.136 (Fisher's exact test)).

### Results by gender

Three-quarters of the sample were female (*n* = 42); 14 were male. Of the 57% (*n* = 8) males tested, 75% (*n* = 6) were deficient or severely deficient in vitamin D, whereas of the 45.2% (*n* = 19) females tested, 79% (*n* = 15) were deficient or severely deficient in vitamin D. There was no statistical association between gender and vitamin D deficiency (χ^2^ = 0.05, *P* = 1.000 (Fisher's exact test)).

## Discussion

### Are adolescents at risk?

There is emerging evidence that the adolescent population in general is at risk of vitamin D deficiency.^8^ Although risk factors may vary, depending on geographical location, seasonal changes, latitude and variations in ultraviolet light levels, there has also been a suggestion of a worldwide re-emergence of rickets in the paediatric population.^9^ The Royal College of Paediatrics and Child Health identifies adolescents as having an ‘increased need’ for vitamin D,^10^ and the draft NICE guidelines^6^ stated that young people undergoing rapid periods of growth are at an increased risk of vitamin D deficiency, although this did not appear in the final guideline.^2^

### Which guidelines to use?

An update to the NICE guidelines regarding vitamin D deficiency was published in November 2014 with the aim of increasing vitamin D supplement use among at-risk groups.^2^ The guidelines include the findings of the *National Diet and Nutrition Survey* showing that 8–24% of children (depending on age and gender) may have vitamin D deficiency (details available from the authors on request). It is also noted that up to 75% of Asian adults may be deficient in vitamin D. The groups at risk of vitamin D deficiency, including adolescents, are:

- people who have low or no exposure to the sun, for example those who cover their skin for cultural reasons, those who are housebound or confined indoors for long periods, and
- people with darker skin, for example people of African, African-Caribbean or South Asian origin.

There appears to be a lack of consensus between various guidelines in defining a biochemical vitamin D deficiency. NICE guidelines^2^ give a level of <25 nmol/L as low vitamin D status, but local laboratory reference ranges and recommendations made in other guidelines vary. For example, the Endocrine Society^4^ and the Society for Adolescent Health and Medicine^7^ provide different recommendations regarding required vitamin D levels, which appear to be higher than those in the NICE guidelines.^2^ Table 2 summarises the guidelines.

**Table 2:** Vitamin D levels by guideline

	Whittington Hospital NHS Trust laboratory interpretation	NICE guidelines	Endocrine Society and SAHM interpretation
< 25 nmol/L	Severe deficiency	Low vitamin D status	Deficiency

25–50 nmol/L	Deficiency	n/i	Deficiency

>50 nmol/L	Minimal levels for bone health	n/i	Recommended or ‘adequate’ level

SAHM, Society for Adolescent Health and Medicine; n/i, no interpretation offered in the guideline.

### Prevention and treatment

Vitamin D deficiency can be treated with oral or intramuscular supplementation, usually in the D_3_ form (cholecalciferol), as this may be more effective than vitamin D_2_ in raising serum 25(OH)D levels.^11^ NICE guidelines outline the daily vitamin D intake requirements to minimise the risk of deficiency in various age groups, with 400 IU for ‘at risk’ adults. Similar requirements have been suggested for adolescents.^8^ Medical causes of vitamin D deficiency, other than reduced sunlight exposure, should also be considered, for example reduced parathyroid hormone levels or malabsorption disorders such as coeliac disease. No single treatment recommendation for vitamin D deficiency is given in national guidelines.

### How much sun exposure is recommended?

A recent NICE guideline, published in February 2016, outlines the risks and benefits of sunlight exposure.^12^ It recognises that advice on sunlight exposure to date has been available from many sources and that the information has often been ‘inconsistent and potentially confusing’.^12^ Young people and their parents/carers could be confused, worrying about balancing advice to limit sun exposure, because of the increase in incidence of skin cancer and other skin disorders, with a need to have sun exposure for vitamin D production.^13,14^ The new NICE guideline acknowledges that, with a proviso that ‘a simple definitive message telling different groups how often and how long they can be exposed to sunlight to ensure minimum risk but maximum benefit’ is not possible because of the multiple biological, social and environmental factors that contribute towards an individual's risk-and-benefit profile.^12^ Specific risk factors for skin cancer such as a family history of the disease should always be considered when giving sun exposure advice, and the NICE guideline also mentions groups who should take ‘extra care to avoid skin damage and skin cancer’. This includes young people.^12^

It is suggested that skin colour charts may be helpful in making judgements about sun exposure advice. Individuals with naturally very light skin (skin types I and II) are at greater risk of sunburn and skin cancer and require shorter times of sun exposure to synthesise vitamin D compared with those with darker skin types (types V and VI), who are at increased risk of vitamin D deficiency in the UK.^12^

Advice should be tailored for the time of year and the time of day. For example, in the UK between March and October and between 11 am and 3 pm, short periods of sun exposure to the forearms, hands or lower legs are required to synthesise vitamin D, whereas longer times are required between 3 pm and 11 am.^12^ Between October and March, there is ‘very little of the ultraviolet B wavelength the skin needs to make vitamin D’.^12^

With so many factors to consider, it is perhaps not surprising that the general advice given in the NICE guideline remains equivocal, with an emphasis on providing consistent, balanced messages regarding the risks and benefits of sunlight exposure for each individual. It is suggested that: ‘short (less than the time it takes for skin to redden or burn), frequent periods of sunlight exposure are best for vitamin D synthesis. In addition, this type of exposure is less likely to result in skin cancer’.^12^ This advice is broadly similar to advice published in a multiagency consensus document in 2010 (https://www.cancerresearchuk.org/sites/default/files/vitamind-consensus.pdf).^7^

### Study recommendations

If it were assumed that all of the individuals in our study who were not tested – deliberately or by accidental omission – had ‘optimum’ vitamin D levels, then 39.3% (*n* = 22) of the total sample had vitamin D deficiency. However, it seems unlikely that all of the non-tested patients had optimal levels given that, for example, 8 BME patients were not tested and 100% of those who were tested were deficient or severely deficient in vitamin D.

Factors influencing whether patients on Simmons House Adolescent Unit were tested for vitamin D before 2015 have not been explored in this project. The apparent idiosyncrasy of testing is beyond the scope of the present study but was one of the reasons for the study and subsequent recommendations detailed in the Appendix.

The results could suggest that all individuals within Simmons House at risk of vitamin D deficiency were identified and their levels tested accordingly. Alternatively, it may be that a proportion of the 52% of individuals not tested had an undetected vitamin D deficiency, particularly those from BME backgrounds given that 100% of the BME patients who were tested had deficiency or severe deficiency. As increased skin pigmentation is identified as a specific risk factor for vitamin D deficiency in the NICE guidelines, it could be that all adolescents with increased skin pigmentation admitted to a tier 4 adolescent psychiatric unit should have their vitamin D levels checked. This may be particularly relevant in units with culturally diverse populations. Specific recommendations made for Simmons House are detailed in the Appendix.

Perhaps any adolescent with a mental illness of a severity that requires in-patient admission is at risk of reduced sun exposure and consequent vitamin D deficiency, no matter their ethnicity. It could be suggested that all patients within tier 4 adolescent psychiatric units should be considered at high risk of vitamin D deficiency and tested, unless there was clear evidence to the contrary, such as a confirmed history of adequate sun exposure and theoretically sufficient dietary intake.

Choosing a particular treatment regime depends on clinical need and consideration of local guidelines. Recommendations given in various London NHS trusts guidelines (St Bartholomew's and The London NHS Trust, Royal Free Hospital NHS Trust and The Whittington Hospital NHS Trust; details available from the authors on request) range from treating a deficiency with oral cholecalciferol in doses of 2000 to 6000 IU per day, or once-weekly doses of 20 000 IU, all for a minimum of 3 months before rechecking vitamin D levels. Once the serum 25(OH)D level has been normalised, the recommended maintenance doses range from 400 to 1000 IU of cholecalciferol per day, with NICE suggesting 400 IU per day as a prophylactic dose for those at risk of deficiency. Therefore, at the very least, it seems that prophylactic supplementation of 400 IU of vitamin D_3_ per day should be considered for adolescents at risk of vitamin D deficiency, or treatment of a confirmed vitamin D deficiency initiated after discussion with the adolescent and/or their family/carers. Treatment regimens should be in line with local guidelines until a sufficient evidence base is established to provide national guidelines.

More research is needed into the prevalence of vitamin D deficiency in all age groups, both in the general population and in hospital settings (general and psychiatric hospitals). The topic is likely to appear in the medical literature frequently in years to come, with hypothesised links between vitamin D deficiency and a multitude of medical conditions ranging from cancer to psychosis,^15^ multiple sclerosis^16^ and the possibility of a worldwide re-emergence of rickets in the paediatric population.^9^ The relationship between vitamin D and mental illness is not known. A meta-analysis^17^ published in the *British Journal of Psychiatry* in 2013 supported an association between low vitamin D concentrations and depression, mostly based on observational studies. However, the nature of the association is not yet known to be causal and the paper also noted that the quality of evidence in this particular area to date is poor; no randomised control trials have been performed.^17^

If an association between depression and vitamin D deficiency were to be confirmed through future study, vitamin D supplementation could potentially be a cost-effective treatment adjunct with minimal adverse effects. In the meantime, the beneficial effects of vitamin D on bone health have been clearly demonstrated. Future research might include a nationwide project through the Royal College of Psychiatrists' Quality Network of Inpatient CAMHS Units (QNIC; qnic.org.uk) to which almost all tier 4 units in the UK are allied for appraisal and accreditation. Additionally, consideration of whether the child and adolescent population attending tier 3 child and adolescent mental health services (CAMHS) should be tested for their vitamin D status requires further thought.

## Appendix Vitamin D recommendations for patients admitted to Simmons House

1. All admissions to Simmons House should be considered at high risk of vitamin D deficiency, especially individuals with increased skin pigmentation or with a history suggestive of a lack of sun exposure. Therefore, vitamin D levels should be included as part of the routine physical assessment.
2. If blood tests are refused or clinically inappropriate, prophylactic treatment should be considered with 400 IU cholecalciferol (vitamin D_3_) orally once daily, assuming informed consent is gained.
3. If a vitamin D deficiency is detected, baseline corrected calcium levels should be tested, plus a full bone mineral profile and testing of parathyroid hormone levels, along with routine admission blood tests (e.g. full blood count, urea and electrolytes, liver function tests, random blood glucose, thyroid function tests and lipid profile).
4. Treatment of a vitamin D deficiency should consist of high-dose cholecalciferol (vitamin D_3_) (5000–6000 IU) orally daily for 3 months. Vitamin D levels and corrected calcium levels should then be checked again and a maintenance dose of cholecalciferol (vitamin D_3_) 400 IU commenced once daily when vitamin D levels have normalised. Continuation of treatment should be tailored to each individual, based on severity of the deficiency and ongoing risk factors for vitamin D deficiency. Longer-term management of supplementation should be discussed with primary care colleagues.
5. Patients and/or their families/carers should be made aware of potential side-effects of treatment i.e. vitamin D toxicity or hypercalcaemia, which may present with anorexia, weight loss, vomiting and polyuria.
6. Ongoing examination of blood test results (serum vitamin D levels) should take place and internal audit on clinical practice should continue.
